# Surgical drain has no benefits in hemiarthroplasty for femoral neck fractures in elderly patients

**DOI:** 10.1038/s41598-023-48799-7

**Published:** 2023-12-05

**Authors:** Seung-Hun Lee, Dae-Kyung Kwak, Je-Hyun Yoo

**Affiliations:** Department of Orthopaedic Surgery, Hallym University Sacred Heart Hospital, Hallym University College of Medicine, 22 Gwanpyeong-ro 170beon-gil, Dongan-gu, Anyang, 14068 South Korea

**Keywords:** Outcomes research, Clinical trial design

## Abstract

Femoral neck fracture is a common osteoporotic fracture in elderly patients and is effectively managed with arthroplasty. However, the benefits and risks of a surgical drain after arthroplasty in these patients are still debatable. Hence, we conducted this study to investigate the necessity of a surgical drain after hemiarthroplasty in elderly patients with femoral neck fracture. This study enrolled elderly patients (aged ≥ 70 years) who underwent cementless bipolar hemiarthroplasty for femoral neck fracture between April 2016 and December 2021. The patients were divided into two groups; the control group (199 patients) with a surgical drain after surgery performed between April 2016 and June 2020 and the study group (134 patients) with no surgical drain between July 2020 and December 2021. The demographics, perioperative data, and postoperative complications were compared between the two groups. Estimated blood loss, perioperative transfusion volume and rate, and length of hospital stay were significantly lower in the study group than in the control group (p < 0.001, p < 0.001, p = 0.008, and p < 0.001, respectively). Although there were no significant intergroup differences in the length of intensive care unit stay and in-hospital, 1-month, and 1-year mortalities, the incidence of postoperative medical complications was significantly lower in the study group than the control group (p = 0.001). A surgical drain may be unnecessary after hemiarthroplasty in elderly patients with femoral neck fracture considering less blood loss and transfusion, lower incidence of postoperative medical complications, and shorter hospital stay in the study group with no surgical drain.

## Introduction

The global population is aging and there has been a significant increase in the incidence of hip fractures in the elderly population^1^. Furthermore, successful surgical treatment of hip fractures in elderly patients is emerging as a key challenge owing to increased morbidity and mortality and functional deterioration^2^. The femoral neck fracture is a common type of hip fractures in the aging population and has been most effectively managed with arthroplasty^3^.

Surgical drain placement after hip arthroplasty has been common practice since Waugh and Stinchfield first recommended suction drainage in 1961^4,5^. However, the benefits and risks of a surgical drain after hip arthroplasty are still controversial. Suction drainage has been applied in hip arthroplasty to decrease hematoma formation and subsequent complications such as infection^6^. Strahovnik et al.^7^ reported various advantages of a surgical drain such as prevention of hematoma and prolonged wound drainage and less mid-thigh pain. However, several studies have demonstrated that there are no statistically significant benefits of a surgical drain and that there is a higher transfusion rate in patients with a surgical drain^5,8–10^. Moreover, Abdel et al.^11^ suggested that a surgical drain should be no longer recommended after hip arthroplasty. Recently, the restrictive use of surgical drainage is a key element introduced by Enhanced Recovery After Surgery (ERAS) protocols^12^. However, these studies were conducted on relatively younger patients who underwent total hip arthroplasty. Therefore, it is difficult to apply these guidelines to fragile elderly patients.

With the aging society, the average age of patients with femoral neck fracture and the severity of their comorbidities are increasing. Despite the improvement of patient management protocols and surgical techniques to reduce perioperative blood loss and transfusion over the past decade, many elderly patients still require perioperative blood transfusion after hip arthroplasty for femoral neck fractures and usually receive allogeneic blood transfusion when there is acute blood loss^13^. However, allogeneic blood transfusion is likely to cause complications such as infection, acute transfusion reaction, transfusion-related circulatory overload, and acute lung injury, especially in fragile elderly patients^14,15^. Meanwhile, complications such as wound problem and subsequent infection that can develop in cases without a surgical drain after hip arthroplasty may be fatal in these patients. However, there is little information on the necessity of a surgical drain after hemiarthroplasty in in fragile elderly patients with femoral neck fracture and its effect on perioperative outcomes.

Therefore, we conducted this retrospective comparative study to investigate the effect of no use of a surgical drain on perioperative outcomes including complications and mortality after cementless hemiarthroplasty in elderly patients with femoral neck fracture, and to evaluate the necessity of a surgical drain in these patients.

## Materials and methods

### Patients

**Figure 1:**
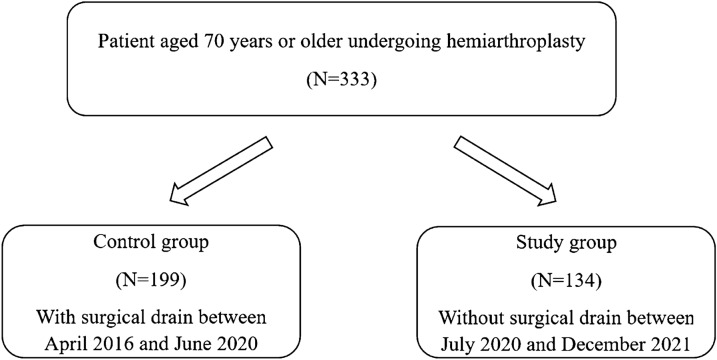
Flowchart demonstrating patient selection.

This retrospective cohort study was approved by the institutional review board of Hallym University Sacred Heart Hospital (2022-10-027). This study was conducted in accordance with the Declaration of Helsinki. The institutional review board waived the informed consent for this study owing to its retrospective nature. All methods were carried out in accordance with the relevant guidelines and regulations. This study enrolled elderly patients (age ≥ 70 years at the time of injury) who underwent cementless bipolar hemiarthroplasty for femoral neck fractures between April 2016 and December 2021 and were followed up for at least 1 year. Finally, 333 patients were identified. Notably, a postoperative surgical drain was used in patients who underwent hemiarthroplasty before July 2020, whereas it was not used in patients from July 2020. The control group comprised 199 patients with a surgical drain after surgery and the study group comprised 134 patients without a surgical drain (Fig. 1).

### Study design

All cementless bipolar hemiarthroplasties were performed using the short external rotators-preserving posterolateral approach by a senior surgeon (JHY) in a single center^16^. After implantation and irrigation, 1 g of tranexamic acid was topically injected into the joint capsule and the soft tissue around the hip joint in all cases. Then, the posterior capsule was repaired. Just before wound closure, a surgical vacuum drain was inserted into the hip joint in the control group. All patients were divided into two groups according to whether a surgical drain was used after surgery.

After surgery, both groups received combined chemical and mechanical thromboprophylaxis until discharge (10–14 days). Low-molecular-weight heparin was used as a chemoprophylactic agent and an intermittent pneumatic compression device and graduated compression stockings were used as mechanical prophylaxis. According to our institutional protocol, patients were instructed to walk under tolerable weight-bearing with an assistive device (walker) from the postoperative day (POD) 2 or 3 depending on patients’ condition. At hospital discharge, it was possible for patients to ambulate with the aid of a walker. Because the National Public Health System and private health insurance companies covered most of the hospitalization cost, post-acute inpatient rehabilitation was performed consecutively during the postoperative hospitalization period. Routine follow-up visits were scheduled at 6 weeks and 3, 6, 9, and 12 months postoperatively, and annually thereafter. Patients or their families were contacted via telephone if the patients did not regularly visit at scheduled times.

The following data were collected from the medical records. Demographic data at the time of surgery included age, gender, body mass index, American Society of Anesthesiologists (ASA) score, comorbidities, and bone mineral density. Preoperative parameters included time to operation, preoperative antiplatelet medication administered for comorbidities, and preoperative hemoglobin (Hb) and hematocrit (Hct) levels. Perioperative data included operation time, anesthesia method, postoperative changes in Hb and Hct levels, the volume of vacuum drainage, transfusion amount and rate, estimated blood loss (EBL), lengths of intensive care unit (ICU) and hospital stay, and ICU admission rate after surgery. Postoperative data included the incidences of venous thromboembolism, delirium, readmission, medical and surgical complications and in-hospital, 1-month, and 1-year mortalities.

Patients received transfusion of packed red blood cells (RBC) when the Hb level decreased below 8 g/dL or at higher levels if a poor general condition, palpitations, dizziness, or pallor was observed.

The EBL was calculated according to the Gross formula, as shown below.

Prediction of blood volume^17^:

$$ \begin{aligned} & {\text{Males:}}\;\;604  +  0.0003668  \times  [{\text{height}}\;({\text{cm}})]^{3} +  32.2  \times {\text{weight}}\;({\text{kg}}) \\ & {\text{Females:}}\;\;183  +  0.000356  \times  [{\text{height}}\;({\text{cm}})]^{3} +  33  \times {\text{weight}}\;({\text{kg}}) \end{aligned} $$

EBL calculation method^18^:

$$ {\text{EBL}} = {\text{blood}}\;{\text{volume}} \times  ({\text{Hct}}_{{{\text{preoperative}}}} {-}{\text{Hct}}_{{{\text{day}}\;{5}\;{\text{postoperative}}}} )  + {\text{volume}}\;{\text{of}}\;{\text{transfused}}\;{\text{RBC}}\;({\text{mL}}). $$

We used Hct levels just before surgery and on POD 5, and the volume of transfused RBC during this period. All transfusions in our cohort were performed during surgery and within POD 3.

### Statistical analysis

Sample size was calculated in G*power with α set as 0.05 and power of 80% (G*Power 3.1.9.4; Heinrich-Heine-Universität Düsseldorf, Düsseldorf, Germany), and it was confirmed to be at least 128 partietns. Statistical analyses were conducted using IBM SPSS, version 24.0 (IBM Corp., Armonk, NY, USA). Student’s *t*-test was used to compare numerical data, which were expressed as mean ± standard deviation. Chi-square and Fisher’s exact tests were used to compare categorical data. Univariate analysis was performed for variables with a p-value < 0.2 in comparison between the two groups to calculate unadjusted crude odd ratio (OR). Finally, multiple logistic regression was performed for the potential confounders with a p-value < 0.1 on univariate analysis and clinically important variables to determine the adjusted OR and p-value. The adjusted ORs were reported with 95% confidence intervals and p-values. Logistic regression analysis for continuous factors was performed using the median values. For all analyses, a p-value of < 0.05 was considered statistically significant.

## Results

**Figure 2:**
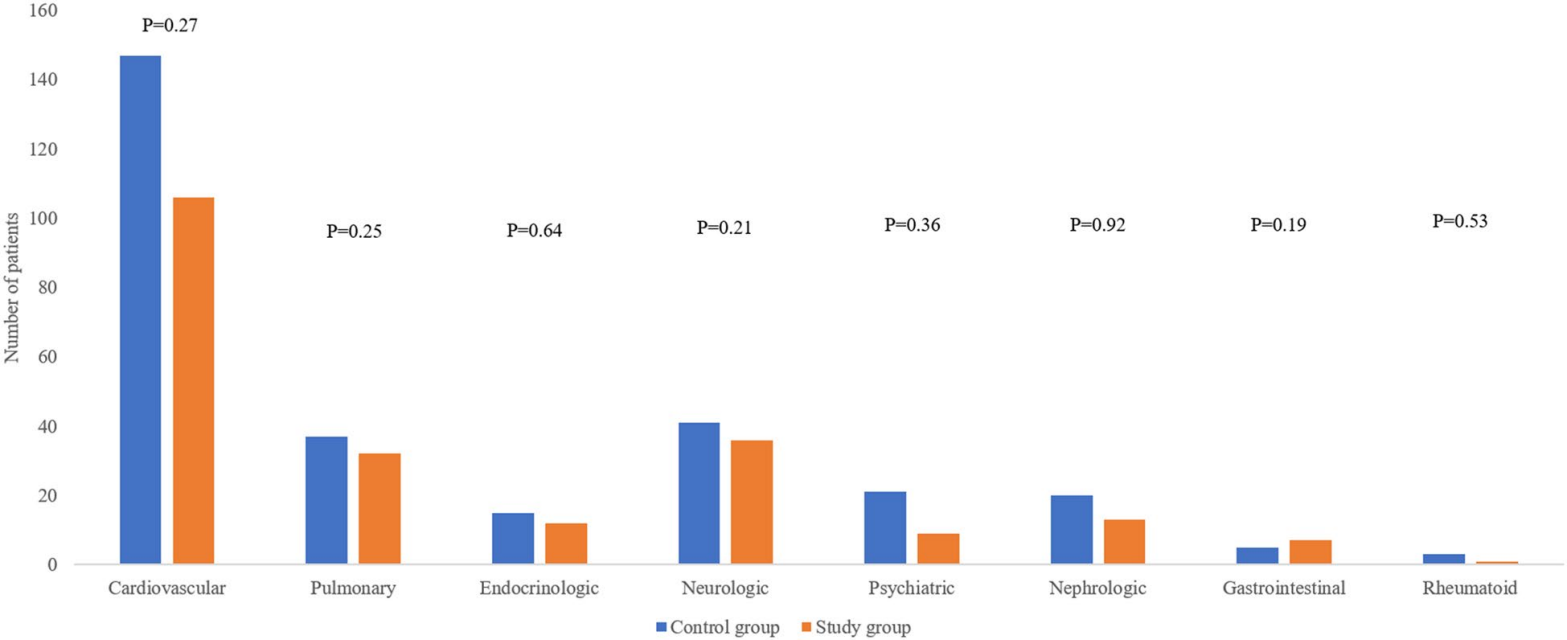
Comparison of comorbid medical diseases between the two groups.

No significant intergroup differences were observed in demographic and preoperative data (Table 1) including comorbid medical diseases (Fig. 2). In terms of perioperative data, there were no significant differences between the two groups in operation time, anesthesia method, immediate postoperative, POD 1, and POD 5 Hb levels, immediate postoperative and POD 5 Hct levels, transfusion rate above Hb 8 g/dL, ICU admission rate, and length of ICU stay (Table 2).

**Table 1:** Demographic and preoperative data in both groups.

	Control group (n = 199)	Study group (n = 134)	*p*-value
Age	82.37 ± 5.80	83.28 ± 5.77	0.160
Gender (male:female)	43:156	26:108	0.628
Body mass index (kg/m^2^)	22.03 ± 3.97	22.39 ± 3.65	0.399
Time to operation (days)	3.01 ± 3.24	3.06 ± 1.95	0.848
ASA score			0.209
II	5	3	
III	163	103	
IV	31	28	
Preoperative antiplatelet medication	82 (41.21%)	60 (44.78%)	0.520
BMD (T-score, femur neck)	− 2.93 ± 1.00	− 2.87 ± 0.81	0.634
Preoperative levels			
Hb (g/dL)	11.58 ± 1.66	11.53 ± 1.93	0.810
Hct (%)	34.76 ± 4.26	34.07 ± 5.36	0.213

*ASA* American Society of Anesthesiologists, *BMD* bone mineral density, *Hb* hemoglobin, *Hct* hematocrit. Continuous variables are presented as mean ± standard deviation.

**Table 2:** Comparison of perioperative data between the two groups.

	Control group (n = 199)	Study group (n = 134)	*p*-value
Operation time (min)	69.74 ± 19.06	70.89 ± 16.77	0.572
Anesthesia (general:spinal)	180:19	122:12	0.856
Immediate postoperative Hb (g/dL)	10.71 ± 1.16	10.93 ± 1.22	0.107
Immediate postoperative Hct (%)	32.23 ± 3.52	32.45 ± 3.59	0.569
Transfusion rate (%)	58.79 (117/199)	44.03 (59/134)	**0.008**
Intraoperative transfusion rate (%)	41.71 (83/199)	36.57 (49/134)	0.348
Postoperative transfusion rate (%)	32.16 (64/199)	16.42 (22/134)	**0.001**
Transfusion rate (%) in Hb > 8 g/dL	5.53 (11/199)	2.24 (3/134)	0.113
POD 1 Hb (g/dL)	10.00 ± 1.16	9.78 ± 1.31	0.102
POD 1 Hct (%)	30.16 ± 3.46	29.00 ± 3.85	**0.004**
POD 5 Hb (g/dL)	9.30 ± 1.08	9.34 ± 1.30	0.750
POD 5 Hct (%)	28.14 ± 3.38	27.74 ± 3.92	0.322
Hospital stay (days)	17.97 ± 9.43	14.67 ± 5.79	**< 0.001**
ICU admission (%)	30.15 (60/199)	35.07 (47/134)	0.347
ICU stay (days)	2.68 ± 3.14	2.72 ± 4.48	0.957

*Hb* hemoglobin, *Hct* hematocrit, *POD* postoperative day, *ICU* intensive care unit. Continuous variables are presented as mean ± standard deviation. Significant values are in bold.

**Figure 3:**
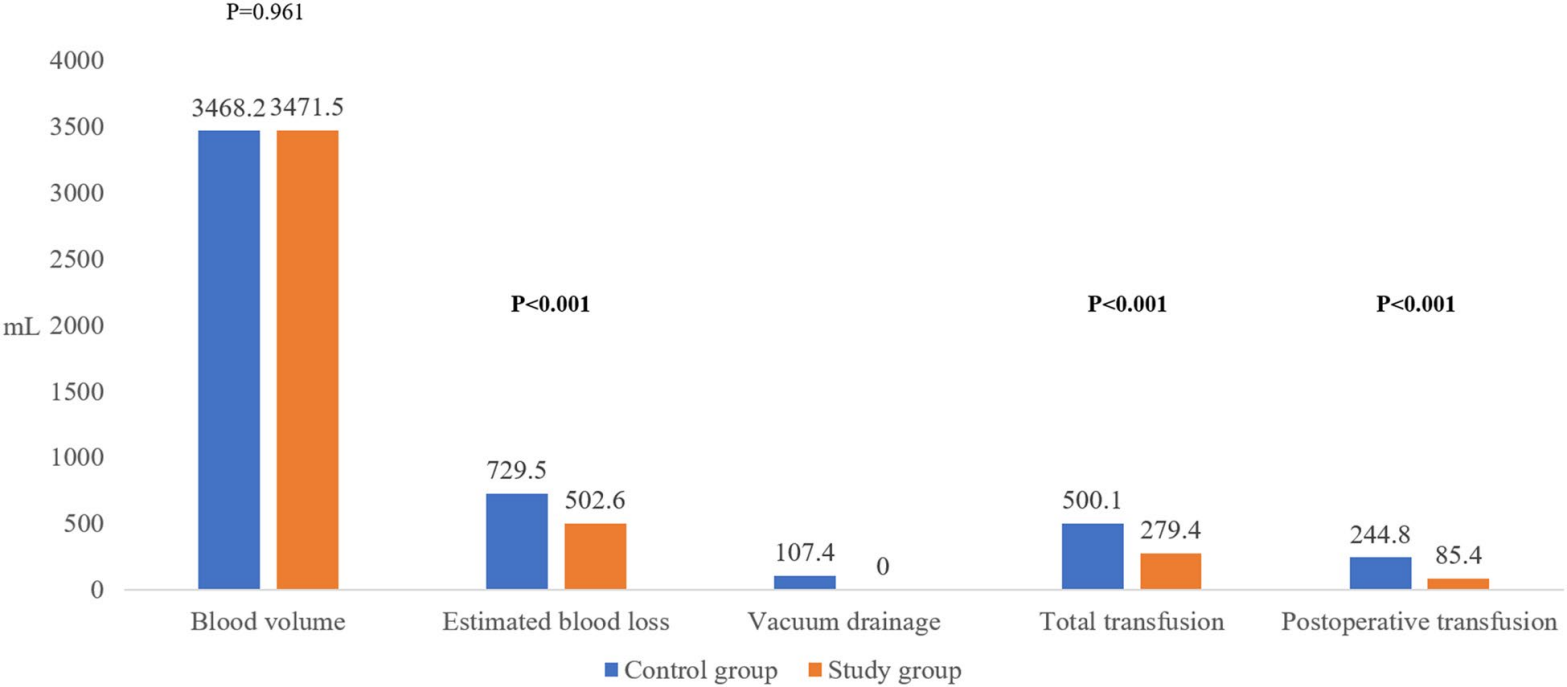
Comparison of perioperative blood loss and transfusion between the two groups.

However, EBL was significantly lower in the study group than in the control group (502.6 ± 343.2 mL vs 729.5 ± 641.9 mL, p < 0.001). Besides, postoperative transfusion volume was significantly lower in the study group than in the control group (244.8 ± 522.7 mL vs 85.4 ± 212.3 mL, p < 0.001) (Fig. 3) and postoperative transfusion rate were significantly lower in the study group (16.4% vs 32.2%, p = 0.001) although there was no significant intergroup difference in transfusion rate above Hb 8 g/dL perioperatively (p = 0.113). The length of hospital stay was significantly shorter in the study group (p < 0.001) (Table 2).

In terms of postoperative complications and mortality, there were no significant intergroup differences in the incidences of deep vein thrombosis, pulmonary embolism, delirium, readmission and in-hospital, 1-month, and 1-year mortality rates. However, postoperative medical complications showed lower incidence in the study group than in the control group (p = 0.001) although there was no significant difference in the incidence of surgical complications such as dislocation, periprosthetic joint infection, and wound infection (Table 3).

**Table 3:** Comparison of postoperative complications between the two groups.

	Control group (n = 199)	Study group (n = 134)	*p*-value
DVT	8 (4.02%)	10 (7.46%)	0.199
PE	5 (2.51%)	8 (5.97%)	0.140
Delirium	62 (31.16%)	48 (35.82%)	0.376
Readmission	5 (2.51%)	1 (0.75%)	0.188
In-hospital mortality	4 (2.01%)	4 (2.99%)	0.570
1-month mortality	5 (2.51%)	4 (2.99%)	0.795
1-year mortality	9 (4.52%)	8 (5.97%)	0.558
Medical complications	94 (47.24%)	40 (29.85%)	**0.001**
Cardiovascular	6	2	
Pulmonary	38	24	
Cerebrovascular	2	4	
Nephrologic	18	4	
Urologic	20	9	
Gastrointestinal	15	2	
Surgical complications	5 (2.51%)	1 (0.75%)	0.188
Dislocation	1	1	
PJI	1	0	
Wound infection	3	0	

*DVT* deep vein thrombosis, *PE* pulmonary embolism, *PJI* periprosthetic joint infection. Significant values are in bold.

On univariate analysis, no surgical drain were significantly associated with lower POD 1 Hct and transfusion rate, less EBL and transfusion volume, fewer medical complications, and shorter length of hospital stay (p < 0.05, Table 4). On multiple logistic regression analysis, ORs and p-values were adjusted for immediate postoperative Hb, POD 1 Hb and Hct, EBL, transfusion rate and volume, medical complications, and length of hospital stay. Finally, EBL, and medical complications was significantly reduced in the study group with no surgical drain (OR = 0.43, p = 0.041; OR = 0.38, p = 0.037, respectively) and the length of hospital stay was significantly shorter in the study group (OR = 0.40, p = 0.042), (Table 4).

**Table 4:** Logistic regression for no surgical drain as an independent variable.

	Univariate analyses Crude OR (95% CI)	*p*-value	Multivariate analyses Adjusted OR* (95% CI)	*p*-value
Immediate postoperative Hb	0.86 (0.78–0.96)	0.110	0.89 (0.76–0.98)	0.252
POD 1 Hb	0.62 (0.41–0.83)	0.122	0.77 (0.56–0.91)	0.387
POD 1 Hct	0.33 (0.14–0.59)	0.007	0.41 (0.22–0.68)	0.122
DVT	2.52 (1.12–3.23)	0.218	1.23 (0.89–1.98)	0.417
PE	1.81 (1.09–7.54)	0.371	1.10 (0.83–4.53)	0.518
Re-admission	0.78 (0.47–0.98)	0.221	0.81 (0.50–0.99)	0.325
EBL	0.23 (0.11–0.42)	0.001	0.43 (0.18–0.91)	**0.041**
Transfusion rate	0.57 (0.26–0.81)	0.006	0.88 (0.45–1.32)	0.211
Transfusion volume	0.36 (0.18–0.85)	0.001	0.84 (0.61–1.07)	0.682
Medical complications	0.12 (0.07–0.36)	0.001	0.38 (0.13–0.74)	**0.037**
Surgical complications	0.89 (0.81–1.03)	0.187	0.91 (0.78–1.23)	0.448
Length of hospital stay	0.13 (0.06–0.42)	0.001	0.40 (0.29–0.81)	**0.042**

*OR* Odds ratio, *CI* confidence interval, *Hb* hemoglobin, *Hct* hematocrit, *POD* postoperative day, *DVT* deep vein thrombosis, *PE* pulmonary edema, *EBL* estimated blood loss.

*Adjusted for immediate postoperative Hb, POD 1 Hb and Hct, EBL, transfusion rate and volume, medical complications, and length of hospital stay. Significant values are in bold.

## Discussion

The use of a surgical drain after hip arthroplasty is still controversial and no uniform guidelines pertaining to this exist, especially for fragile elderly patients. Cheung et al.^19^ suggested that no drain is required after total hip replacement surgery owing to the significantly shorter length of hospital stay, quicker wound drying, and economic savings. Zhou et al.^20^ reported that a surgical drain reduces the requirement for dressing reinforcement, but increases the rate of perioperative blood transfusion. In a meta-analysis of randomized controlled trials, Chen et al.^21^ reported that the use of closed suction drainage in hip arthroplasty increased the requirement for postoperative blood transfusion, but there were no significant differences in the incidence of postoperative hematoma, wound dehiscence, or deep vein thrombosis. However, these studies were conducted on relatively younger patients who underwent elective total hip or knee arthroplasty. We believe that these results cannot be applied consistently to fragile elderly patients undergoing hemiarthroplasty for femoral neck fractures. Therefore, this retrospective comparative study was conducted to investigate the effect of no surgical drain on perioperative outcomes including medical and surgical complications and mortality after hemiarthroplasty in fragile elderly patients with femoral neck fracture, and to evaluate the necessity of a surgical drain in these patients.

Compared with patients undergoing total hip arthroplasty, elderly patients undergoing hemiarthroplasty for femoral neck fractures are generally more fragile and have multiple comorbidities. Also, most of these patients have cardiovascular or endovascular diseases receiving subsequent antiplatelet medication. These patients are more vulnerable to perioperative blood loss and transfusion, hematoma formation, wound infection, periprosthetic joint infection, or medical complications^22^. These problems are directly or indirectly related to short-term and long-term mortalities and it is of paramount importance to reduce perioperative blood loss and transfusion requirements in these elderly patients^23^. However, there are few studies reporting the effect of a surgical drain on perioperative outcomes including postoperative complications and mortality in elderly patients who underwent hemiarthroplasty for femoral neck fractures. In fragile elderly patients, more caution regarding perioperative blood loss, transfusion, and subsequent postoperative complications is required for better outcomes. The reduction in perioperative blood loss and transfusion may directly or indirectly reduce the length of hospital stay and postoperative medical complications in these patients, as shown in the current study.

Our study revealed that no use of a surgical drain reduces perioperative blood loss, transfusion rate and volume, and medical complications in elderly patients who underwent hemiarthroplasty for femoral neck fractures. It would be difficult to establish that fewer medical complications resulted from reduced blood loss and transfusion due to no use of a surgical drain. However, no use of a surgical drain and subsequent less blood loss and perioperative transfusion might have accelerated ambulation and rehabilitation in these patients compared to those with a surgical drain after surgery, who subsequently received more blood transfusions due to greater blood loss. Besides, no use of a surgical drain could subsequently shorten the hospital stay. Fishman et al.^24^ found a significantly shorter hospital stay for patients without a surgical drain (4.3 days without a drain, versus 5.4 days with a drain, p = 0.002). A shorter hospital stay may also lower the risk of postoperative medical complications.

Currently, only two studies have reported the effect of a surgical drain after hemiarthroplasty in geriatric patients with femoral neck fracture^10,25^. One study reported only 86 patients with no clinically significant differences bewteen patients with and without a surgical drain^10^. Meanwhile, the other study reported that surgical drain placement decreased postoperative wound hematoma, but required longer hospital stay along with more Hb loss and greater transfusion requirements during admission after hemiarthroplasty for femoral neck fractures^25^. Similarly in our study, they demonstrated the benefits of no surgical drain rather than the disadvantages. Although their study included sufficient patients to draw significant conclusions, there were significant differences in baseline characteristics such as age and general condition status (ASA score) between the two groups with and without a drain, and they did not consider multiple comorbidities of patients enrolled in both groups. We believe that these patient-related factors are very important because they may affect clinical outcomes in this comparative study and the heterogeneity of the two groups weakens the reliability of their conclusions. In the present study, the two groups were homogeneous in terms of demographic data including age, gender, general condition status, and comorbidities. Therefore, we believe that our study provides more reliable clinical evidence to decide whether to use a surgical drain after hemiarthroplasty in fragile elderly patients with femoral neck fracture and reveals that a surgical drain is not necessary in these patients.

In the current study, the total transfusion rate was relatively higher than in other studies reporting a 14 to 26% rate^26,27^. We think that the reasons could be as follows; the enrolled patients who underwent hemiarthroplasty were older than those who underwent elective total hip arthroplasty in other studies and most of them (97.6%) had ASA score of ≥ 3, who were subsequently vulnerable to perioperative blood loss. Therefore, more patients tended to receive blood transfusion even at Hb > 8 g/dL according to the recommendations of the anesthesiologists intraoperatively and the internal medicine specialists postoperatively, considering the patients’ comorbidities and medical conditions. In particular, elderly patients with a high risk of complications due to chronic diseases were more likely to receive intraoperative blood transfusions. However, there was no significant difference in the perioperative transfusion rate at Hb > 8 g/dL between the two groups.

However, this study also had the following limitations. First, this was a retrospective study with a relatively small sample size despite the use of prospectively compiled data. Second, we could not consistently control allogenic blood transfusion in all patients since most perioperative transfusions were performed according to the recommendations of anesthesiologists or internal medicine specialists considering the patients’ comorbidities and medical conditions in fragile elderly patients, especially those with Hb > 8 g/dL. Third, the duration of a surgical drain and the negative drain pressure were not consistently maintained. Finally, the incidence of thromboembolism was not assessed accurately, as only patients with clinical symptoms were subjected to diagnostic tests, such as ultrasonography or 3-dimensional computed tomography-angiography.

Despite these limitations, to the best of our knowledge, this is the first single-center study to evaluate the effect of not using surgical drain on perioperative outcomes including postoperative complications and mortality in fragile elderly patients undergoing hemiarthroplasty for femoral neck fractures. Both groups underwent the procedures performed by the same surgeon using the same approach. Besides, both groups showed a homogeneity with no significant differences in demographic and preoperative data. Therefore, whether to use a surgical drain after hemiarthroplasty was the sole independent variable between the two groups. Moreover, there was no patient- selection bias since whether to use a surgical drain depended only on the period of the surgery. Finally, our assessment of the amount of total blood loss based on the Hct levels and Gross formula was more accurate than assessments based on clinical calculation involving blood-soaked gauze, suction bottles, and vacuum drains. However, our results will have to be substantiated by further large multi-center prospective studies.

In conclusion, the present study showed that no use of a surgical drain significantly reduced perioperative blood loss, transfusion rate and volume, and medical complications along with shortened hospital stay in fragile elderly patients who underwent cementless hemiarthroplasty for femoral neck fractures. These results suggest that a surgical drain may be unnecessary after hemiarthroplasty in these patients. In the context of the increase of elderly patients with hip fracture worldwide, we believe that our study provides sound clinical evidence of no surgical drain for better surgical outcomes in these patients.

## Data Availability

Data is available from the corresponding author on reasonable request.
